# Seroprevalence for *Borrelia burgdorferi* sensu lato and tick-borne encephalitis virus antibodies and associated risk factors among forestry workers in northern France, 2019 to 2020

**DOI:** 10.2807/1560-7917.ES.2023.28.32.2200961

**Published:** 2023-08-10

**Authors:** Alexandra Septfons, Emma Rigaud, Laetitia Bénézet, Aurelie Velay, Laurence Zilliox, Lisa Baldinger, Gaëlle Gonzalez, Julie Figoni, Henriette de Valk, Gaëtan Deffontaines, Jean Claude Desenclos, Benoit Jaulhac

**Affiliations:** 1 Santé publique France, Saint-Maurice, France; 2 Caisse Centrale de la Mutualité Sociale Agricole, Bobigny, France; 3 Virology Laboratory, University Hospital of Strasbourg, Strasbourg, France; 4 French National Reference Center for Borrelia, University Hospital of Strasbourg, Strasbourg, France; 5 ANSES, INRAE, Ecole Nationale Vétérinaire d'Alfort, UMR VIROLOGIE, Laboratoire de Santé Animale, Maisons-Alfort, France; 6 Institut de Bactériologie, Fédération de Médecine Translationnelle de Strasbourg, University of Strasbourg, Strasbourg, France; * These authors contributed equally to the work and share first authorship.

**Keywords:** *Borrelia burgdorferi*, Lyme borreliosis, seroprevalence, forestry workers, tick, tick-borne encephalitis, tick-borne encephalitis virus

## Abstract

**Background:**

Lyme borreliosis (LB) is the most common tick-borne disease (TBD) in France. Forestry workers are at high risk of TBD because of frequent exposure to tick bites.

**Aim:**

We aimed to estimate the seroprevalence of *Borrelia burgdorferi* sensu lato and tick-borne encephalitis virus (TBEV) antibodies among forestry workers in northern France. We compared seroprevalence by geographical area and assessed factors associated with seropositivity.

**Methods:**

Between 2019 and 2020, we conducted a randomised cross-sectional seroprevalence survey. *Borrelia burgdorferi* sl seropositivity was defined as positive ELISA and positive or equivocal result in western blot. Seropositivity for TBEV was defined as positive result from two ELISA tests, confirmed by serum neutralisation. We calculated weighted seroprevalence and adjusted prevalence ratios to determine association between potential risk factors and seropositivity.

**Results:**

A total of 1,778 forestry workers participated. Seroprevalence for *B. burgdorferi* sl was 15.5% (95% confidence interval (CI): 13.9–17.3), 3.5 times higher in the eastern regions than in the western and increased with seniority and with weekly time in a forest environment. Seroprevalence was 2.5 times higher in forestry workers reporting a tick bite during past years and reporting usually not removing ticks rapidly. Seroprevalence for TBEV was 0.14% (95% CI: 0.05–0.42).

**Conclusion:**

We assessed for the first time seroprevalence of *B. burgdorferi* sl and TBEV antibodies among forestry workers in northern France. These results will be used, together with data on LB and tick-borne encephalitis (TBE) incidence and on exposure to tick-bites, to target prevention programmes.

**Key public health message**

**What did you want to address in this study?**

*Borrelia burgdorferi* sensu lato (sl), bacteria causing Lyme borreliosis, and tick-borne encephalitis (TBE) virus are transmitted to humans via tick bites. We wanted to investigate these infections in forestry workers in northern France and map high risk areas for tick-borne diseases (TBD). We also aimed to study factors associated with these infections, knowledge about TBD and attitudes and practices on preventive measures against tick bites.

**What have we learnt from this study?**

One in six forestry workers had been infected with *B. burgdorferi* sl, but infection with TBE virus was very rare. Past infection by *B. burgdorferi* sl was widespread but forestry workers in the eastern regions of northern France were at highest risk. Forestry workers knew about ticks and TBD and are more likely to protect themselves than the general population. Rapid tick removal appears to protect against infection with *B. burgdorferi* sl.

**What are the implications of your findings for public health?**

Since better information on the risk of TBD can lead to better compliance with preventive measures, communication and preventive campaigns should be continued for professionals at high risk of tick bites, but also for the general population living in or visiting areas with high risk of TBD. The results of this study will be used, with data on the incidence of TBD, to map these risk areas.

## Introduction

Lyme borreliosis (LB) is the most common tick-borne disease (TBD) in France and Europe. It is caused by bacteria of the *Borrelia burgdorferi* sensu lato (sl) complex and transmitted to humans via a bite of infected *Ixodes* ticks [1]. The disease can be treated with antibiotics. Tick-borne encephalitis (TBE), caused by the tick-borne encephalitis virus (TBEV) and transmitted by *Ixodes* ticks, is the most common arboviral disease affecting the human central nervous system in Europe and north-eastern Asia [2]. In France, vaccination against TBE is only recommended for people travelling to countries with highly endemic areas. *Ixodes* ticks are present in almost all of France, especially in wooded and humid areas [3].

In France, surveillance of LB is based on a combination of a nationwide sentinel network of general practitioners (GPs) and analysis of the national hospitalisation discharge database [4,5]. The incidence of GP consultations for LB increased between 2009 and 2020 from 46 to 91 LB cases per 100,000 inhabitants [4]. Lyme borreliosis has been diagnosed in every region, but the incidence is substantially greater in the eastern and central regions of France. The prevalence of *Ixodes* nymphs (responsible for most transmissions) infected with *B. burgdorferi* sl varies between regions: 2% in the west, 10% in the Ile-de-France, 18% in Alsace and 18% in Auvergne [6-10].

In Europe, the incidence of TBE has been increasing [11] and expanding geographically during the past decades [12-14]. In France, approximately 10 cases are reported each year, mainly from the Alsace region (north-eastern France). Since 2003, one or two cases a year have been reported in the French Alpine region [14]. In 2020, an outbreak of TBE in the Rhône-Alpes region was linked to the consumption of raw cheese [15]. Since 2021, cases of TBE are mandatorily notifiable.

Forestry workers are at high risk of LB and other TBD because of frequent occupational exposure to tick-infested environments [16-18] and thus to tick bites [19]. In France, some seroprevalence studies have been conducted regionally, but they do not give a comprehensive view of the distribution of the exposure to tick-borne pathogens [20,21].

We chose to study *B. burgdorferi* sl in forestry workers in the northern part of France as these persons are highly exposed to ticks and tick bites. From an occupational health perspective, we aimed to estimate the proportion of forestry workers infected with *Borrelia burgdorferi* sl and TBEV, to assess knowledge, attitudes and practices among forestry workers related to ticks and tick-borne diseases and to raise awareness on tick-borne diseases. Also, we studied forestry workers as sentinels to indicate high risk areas and to identify and assess factors associated with seropositivity. In addition, we compared seroprevalence by geographical area. Forestry workers in France have the same occupational health scheme, with regular consultations with an occupational physician, which enabled a structured comparison.

We wanted to use this gathered information to adapt communication campaigns to forestry workers and to the general public.

## Methods

### Study design and population

**Figure 1:**
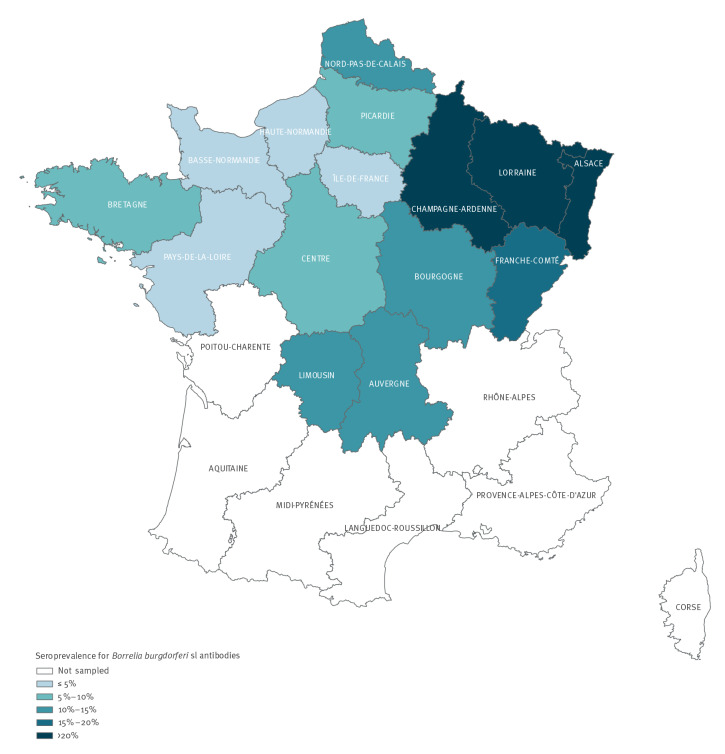
Estimated prevalence of *Borrelia burgdorferi* sensu lato antibodies in forestry workers by region, northern France, 2019–2020

We conducted a random cross-sectional seroprevalence survey in northern France from May 2019 to March 2020 (date of the first lockdown in France due to the COVID-19 pandemic). The target population consisted of all forestry workers aged 18 years or older and monitored by occupational health physicians of the Agricultural Social Fund (Mutualité Sociale Agricole (MSA) in 15 administrative regions of France (n=11,009) (Figure 1). Based on previous knowledge on the epidemiology of Lyme borreliosis with higher reporting rates of Lyme borreliosis in the northern half of France and because of limited resources, we performed this study in the northern half of the country.

### Sample size calculations

The number of study participants needed was calculated for five geographical areas: eastern area, central area, and western area and the administrative Limousin and Auvergne regions. Assuming a *B. burgdorferi* sl seroprevalence of 20% in Limousin region, 10% in Auvergne region, 15% in the eastern area and 5% in the central and western areas [4,20], 2,591 individuals were required to achieve a precision between 1.5 and 4% for the estimation of *B. burgdorferi* sl seroprevalence. Considering the usual participation rate of forestry workers invited for MSA occupational follow-up visits (40–80% depending on the form of employment), 4,484 individuals had to be contacted to obtain the required sample size.

### Sampling design

The sampling frame was constituted from the administrative files of the local MSA occupational health services and included adults working in the forestry sector for at least 120 days in an administrative region covered by an occupational physician that agreed to participate in the survey (n = 10,547).

We used a stratified random sample design. Strata were defined by region, occupation and form of employment (self-employed, employee, other). The number of participants needed in each region was determined in proportion to the size of each region within the geographical area and to achieve the required sample size. The number of individuals sampled was proportional to the size of each stratum. Within each stratum, a simple random sampling was used to select individuals.

### Data collection and blood sampling

After obtaining an informed written consent, the participants were interviewed using a standardised questionnaire and blood samples were collected. The questionnaire was in French, with open-ended or multiple-choice questions on sociodemographic factors (age, sex, place of residence, nationality), professional activities during the past 12 months, exposure to tick bites, leisure activities in the forest, history of erythema migrans, foreign travel during the past 20 years, past vaccination against TBE, yellow fever and Japanese encephalitis and the use of preventive measures against tick bites.

### Data analysis

We present weighted seroprevalence estimates for the whole population and stratified by socio-demographic variables and exposure to ticks.

Prevalence ratios and 95% confidence intervals (CI) were estimated using weighted Poisson regression to assess the association between potential risk factors and *B. burgdorferi* sl seropositivity.

Variables associated with seropositivity with a p value ≤ 0.20 in univariate analysis were included in the multivariate model. Stepwise multivariate Poisson regression was used to investigate independent risk factors for seropositivity. A p value < 0.05 was considered statistically significant.

The weights took into account the sampling weight and were adjusted for non-response. Non-response was corrected by reweighting using the equal-quantile score method [22], based on sociodemographic and geographical data, employment form (self-employed, employee, other) and occupation available in the sampling frame. A calibration by raking ratio method was then applied, using the distributions by sex, age group, employment form and occupation in the target population, using the SAS macro Calmar [23].

Statistical analyses were performed with Stata14 (StataCorp LP, Texas, United States).

### Laboratory methods

**Figure 2:**
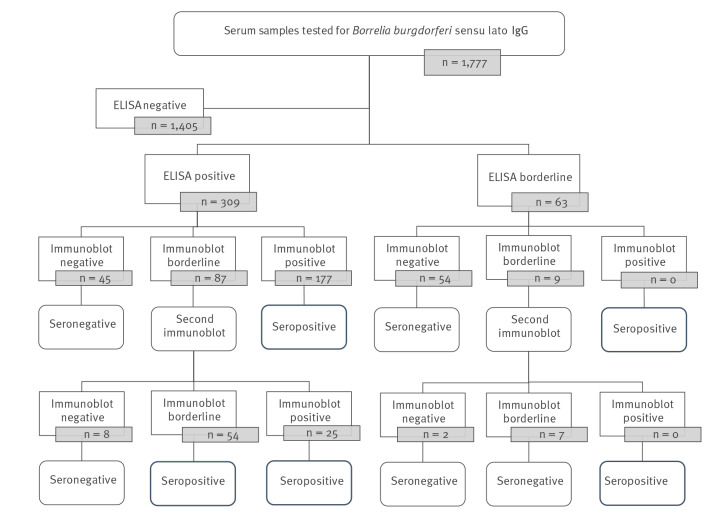
Flowchart of the serological analysis of *Borrelia burgdorferi* sensu lato, France, 2019–2020 (n = 1,777)

Serum samples were tested for *B. burgdorferi* sl-specific IgG antibodies using two-tiered serodiagnostic testing: all samples were first analysed by ELISA (Enzygnost Lyme link VlsE/IgG, Siemens). Specificity and sensitivity of this IgG ELISA for Lyme disease disseminated manifestations was 91% and ≥ 97%, respectively. Samples with positive or borderline results in ELISA were then tested by IgG immunoblot (immunoblot Borrelia Europe LINE plus TpN17 IgG INGEN, Virotech). Specificity and sensitivity of this immunoblot for Lyme disease disseminated manifestations was ≥ 98% and ≥ 97%, respectively. Samples with borderline results in immunoblot were retested using the same immunoblot. To categorise samples by test results, we applied the algorithm shown in Figure 2.

**Figure 3:**
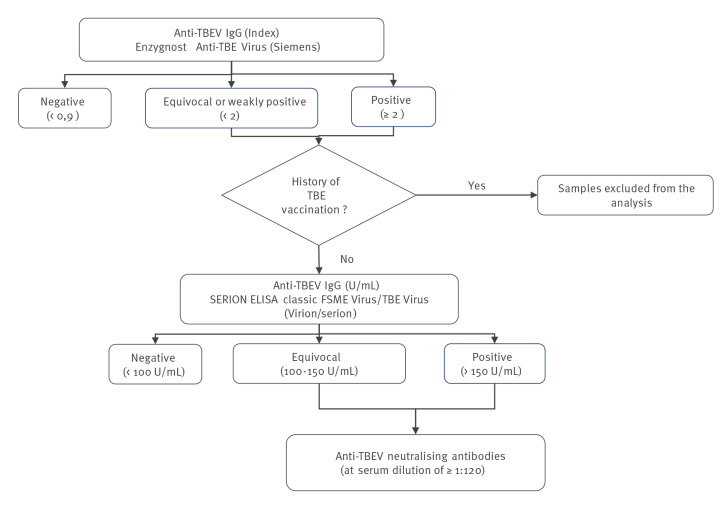
Flowchart of the serological analysis of tick-borne encephalitis virus, France, 2019-2020 (n = 1,777)

FSME: Frühsommer-Meningoenzephalitis; TBE: tick-borne encephalitis; TBEV: tick-borne encephalitis virus; U: unit.

Serum samples were also tested for the presence of TBEV-specific IgG antibodies using the ELISA diagnostic kit Enzygnost Anti-TBE Virus (Siemens). Participants with a history of TBEV vaccination were excluded from the analysis. Anti-TBEV IgG positive and equivocal results were first confirmed using the SERION ELISA classic Frühsommer-Meningoenzephalitis (FSME) Virus/TBE Virus IgG kit (Virion\Serion GmbH, Würzburg, Germany). These two ELISAs previously showed good analytical performances (sensitivity of 100% and specificity 95.9–98.1% for IgG) and a good overall agreement for IgG (Kappa value: 0.94) [24]. Anti-TBEV IgG results assigned as positive or equivocal combining the two serological tests were then confirmed by a serum neutralisation test to exclude any cross-reactivity with other flavivirus infections (strain Hypr, GenBank ID U39292.1) [25]. A serum sample was considered positive for TBEV if the cells were protected at least at the serum dilution of 1:120 (Figure 3).

## Results

A total of 1,778 forestry workers participated in the study, resulting in a response rate of 41% [26]. Serological test results were available from 1,777 participants.

The forestry workers were predominantly male (94.1%), of French nationality (97.3%) and the median age was 45 years (range: 18–87).

We present weighted seroprevalence estimates. A total of 78.4% (95% CI: 76.3–80.3) forestry workers spent time in forests more than 20 hours per week. Most forestry workers (90.5%; 95% CI: 89.1–91.8), reported a history of tick bites and 69.3% of them (95% CI: 66.9–71.6) had been bitten during the last 12 months. Among the latter, 8.4% (95% CI: 6.8–10.2) had noticed a red skin rash around the bite in the weeks following the bite. The proportion of forestry workers bitten by a tick increased with the length of employment in the sector: 86.0% (95% CI: 81.2–89.5) of the individuals with less than 5 years of experience to 95.3% (95% CI: 92.5–97.0) of those who had worked for more than 30 years (p < 0.001). Eighty-eight per cent (95% CI: 86.3–89.3) of forestry workers felt exposed to tick bites during their professional activities, 56.0% (95% CI: 53.4–58.1) very exposed.

Most forestry workers reported the use of protective clothing (62.4%; 95% CI: 60.1–64.7) and 25.8% (95% CI: 23.7–27.9) the use of repellent. Body checks for ticks after forest exposure were usually performed by 74.3% (95% CI: 72.2–76.3) and 97.9% (95% CI: 97.2–98.8) reported usually removing any ticks promptly. Ninety-two per cent (95% CI: 90.7–93.2) were aware of TBD and 77.9% (95% CI: 75.9–79.8) felt well informed about LB.

### *Borrelia burgdorferi* sensu lato antibodies

Anti-*Borrelia* IgG antibodies were detected in 256 of the forestry workers, thus leading to an overall seroprevalence of 15.5% (95% CI: 13.9–17.3).

Forestry workers reporting between 13 and 24 or more than 24 tick bites during the preceding year (22.8%; 95% CI: 16.3–30.9 and 26.9%; 95% CI: 19.9–35.4) were more often seropositive compared with those not bitten (11.4%; 95% CI: 8.9–14.6), p < 0.001 Table 1). Moreover, seroprevalence was 2.5 times higher in forestry workers who reported a tick bite during the past year and who did not usually remove the ticks within 24 h (Table 2).

**Table 1:** Estimated seroprevalence of *Borrelia burgdorferi* sensu lato among forestry workers by epidemiological characteristics, northern France, 2019–2020

Characteristics	Participants n = 1,778^a^	Positive	Weighted seroprevalence		p value
		n	%	95% CI	
Sex					
Male	1,715	254	16.3	14.6–18.1	0.006
Female	63	2	3.7	1.0–12.5	
Age (years)					
18–30	326	18	5.1	3.3–8.0	< 0.001
31–40	377	32	8.9	6.3–12.3	
41–50	430	39	9.9	7.4–13.2	
51–60	520	126	26.6	22.9–30.6	
> 60	125	41	35.8	28.0–44.3	
Type of occupation (nine missing values)					
Forest machine operator	320	32	11.8	8.5–16.2	< 0.001
Forest technician/ranger	357	80	23.1	19.1–27.7	
Gardener/landscaper	20	2	13.1	3.8–36.8	
Hunting technology/gamekeeper/ fishery guardian	144	12	8.6	5.1–14.2	
Silviculturist	289	42	17.0	12.9–22.0	
Woodcutter	622	86	14.1	11.6–17.0	
Other	17	1	3.2	0.5–17.3	
Geographical area of work^b^					
Eastern	673	148	21.3	18.5–24.3	< 0.001
Central	401	39	8.6	6.4–11.3	
Western	396	21	5.5	3.7–8.0	
Auvergne	164	27	15.3	10.9–21.0	
Limousin	144	21	14.4	9.8–20.6	
Nationality (72 missing values)					
French	1,659	241	15.8	14.1–17.7	0.02
Other EU countries	47	2	4.5	1.2–14.7	
Number of years in forestry sector					
1–5	241	16	7.0	4.3–11.1	< 0.001
6–10	242	13	5.0	3.0–8.3	
11–20	449	37	8.3	6.1–11.2	
21–30	406	68	18.2	14.6–22.3	
31–40	317	85	28.8	24.1–33.9	
> 40	123	37	35.0	27.1–43.7	
Average weekly time in forest (hours) (19 missing values)					
< 10	160	13	7.3	4.4–12.0	< 0.001
10–20	199	32	16.4	11.9–22.2	
> 20	1,400	207	16.4	14.5–18.4	
Tick bites (28 missing values)					
No	182	13	7.6	4.6–12.3	0.002
Yes	1,568	236	16.2	14.4–18.1	
If Yes, time from the last tick bite (one missing value)					
< 1 year	1,072	186	18.5	16.3–21.0	< 0.001
> 1 year	495	50	10.8	8.4–13.9	
If Yes, number of tick bites during the last year (five missing values)					
0	494	52	11,4	8.9–14.6	< 0.001
1–12	809	127	16.9	14.4–19.7	
13–24	131	30	22.8	16.3–30.9	
> 24	129	32	26.9	19.9–35.4	
If bitten last year, have you seen a red rash around the bite? (nine missing values)					
No	963	161	18.1	15.7–20.9	0.5
Yes	91	21	22.3	15.0–31.9	
Do not know	9	3	28.1	8.7–61.6	
Exposed to tick bites (33 missing values)					
No	220	16	7.9	5.0–12.3	< 0.001
Yes	1,525	233	16.4	14.6–18.4	
Area of LB incidence^c^					
Low	266	13	4.4	2.5–7.6	< 0.001
Medium	608	65	10.7	8.6–13.3	
High	904	178	19.9	17.5–22.5	

CI: confidence interval; EU: European Union; LB: Lyme borreliosis.

^a^ One blood sample could not be analysed.

^b^ Eastern includes Alsace, Lorraine, Champagne-Ardenne, Bourgogne and Franche-Comté regions; central includes Nord-Pas-De-Calais, Picardie, Île-de-France, Haute-Normandie and Centre regions; western includes Basse-Normandie, Bretagne and Pays-de-la-Loire regions.

^c^ Incidences estimated by the sentinel network in 2018: low < 50 cases per100,000 inhabitants, medium 50–100 cases per 100,000 inhabitants, high > 100 cases per 100,000 inhabitants.

**Table 2:** Factors associated with seropositivity for *Borrelia burgdorferi* sensu lato antibodies in forestry workers, uni- and multivariate analysis, northern France, 2019–2020

Characteristics	Univariate analysis				Multivariate analysis		
	Prevalence ratio	95% CI	p value	Overall p value	Prevalence ratio	95% CI	p value
Type of occupation in forestry (nine missing values)							
Silviculturist	1.2	0.9–1.7	0.272	< 0.001	1.4	1.1–1.9	0.017
Woodcutter	Reference				Reference		
Forestry machine operator	0.8	0.6–1.2	0.357		0.9	0.6–1.3	0.606
Forest technician/ranger	1.6	1.3–2.2	< 0.001		1.4	1.1–1.8	0.019
Hunting technology/gamekeeper/fishery guardian	0.6	0.4–1.1	0.08		0.8	0.5–1.4	0.505
Gardener/Landscaper	0.9	0.3–3.0	0.905		2	0.7–5.2	0.171
Other	0.2	0.04–1.4	0.105		0.3	0.04–2.0	0.203
Geographical area of work^a^							
Eastern	3.9	2.6–5.9	< 0.001	< 0.001	3.5	2.3–5.3	< 0.001
Central	1.6	1.0–2.5	0.07		1.6	1.0–2.6	0.062
Western	Reference				Reference		
Auvergne	2.8	1.7–4.6	< 0.001		3.2	1.9–5.4	< 0.001
Limousin	2.6	1.5–4.5	< 0.001		2.7	1.6–4.7	< 0.001
Average weekly time in forest (hours), (19 missing values)							
< 10	Reference			< 0.001	Reference		
10–20	2.2	1.2–4.1	0.008		1.8	1.0–3.2	0.059
> 20	2.2	1.3–3.8	0.002		2.1	1.2–3.5	0.007
Number of years in forestry sector							
1–5	Reference			< 0.001	Reference		
6–10	0.7	0.4–1.4	0.343		0.7	0.3–1.4	0.292
11–20	1.2	0.7–2.1	0.558		1.2	0.7–2.1	0.492
21–30	2.6	1.5–4.3	< 0.001		2.6	1.6–4.4	< 0.001
31–40	4.1	2.5–6.8	< 0.001		3.8	2.3–6.2	< 0.001
> 40	5	2.9–8.4	< 0.001		5.1	3.1–8.6	< 0.001
Having been bitten and usually removed ticks < 24 h (50 missing values)							
No	4.5	2.0–10.1	0.004	< 0.001	2.5	1.1–5.4	0.023
Yes	2.1	1.3–3.4	< 0.001		1.4	0.9–2.2	0.191
Not bitten	Reference				Reference		

CI: confidence interval.

^a^ Eastern includes Alsace, Lorraine, Champagne-Ardenne, Bourgogne and Franche-Comté; Central includes Nord-Pas-De-Calais, Picardie, Île-de-France, Haute-Normandie and Centre; Western includes Basse-Normandie, Bretagne and Pays-de-la-Loire.

A higher seroprevalence was observed in persons exposed to tick bites (16.4%; 95% CI: 14.6–18.4) and in those living in medium or high endemic areas for LB (10.7%; 95% CI: 8.6–13.3) and 19.9%; 95% CI: 17.5–22.5, respectively) compared with those not exposed to ticks or living in low endemic areas (Table 1).

A west-east gradient for *B. burgdorferi* seroprevalence was observed (Figure 1), ranging from 5.5% in the western region to 21.3% in the eastern region, corresponding to a prevalence ratio of 3.5 between these two regions (Table 2).

The proportion of seropositive forestry workers increased with duration of employment: from 7.0% (95% CI: 4.3–11.1) in persons with 1–5 years of work experience in the sector to 35.0% (95% CI: 27.1–43.7) in those with over 40 years (Table 1). Older forestry workers were more often seropositive than younger ones (from 5.1% (95% CI: 3.3–8.0) in the age group 18–30 years to 35.8% (95% CI: 28.0–44.3) in those over 60 years) (Table 1). Thus, the proportion of seropositives was 2.6 times higher among forestry workers with 20–30 years of experience in the sector and 5.1 times higher among those working more than 40 years compared with those with less than 5 years of work in the sector (Table 2).

Seropositivity for *B. burgdorferi* sl was also associated with the occupation. The proportion of seropositive persons was 1.4 times higher for silviculturists and for forest technicians compared with woodcutters (Table 2). Seroprevalence increased with the average weekly worked hours in a forest environment, persons working more than 20 h in a forest environment had two times higher seroprevalences than those working less than 10 h (Table 2).

### TBEV antibodies

Among the 1,777 forestry workers from whom samples were available for analysis, 26 tested positive or equivocal for anti-TBEV antibodies. Among those, 10 were excluded because of past TBE vaccination. Serum neutralisation test confirmed the presence of specific anti-TBEV antibodies in three forestry workers. The remaining 13 persons had antibodies to other flaviviruses, either from previous travels and/or yellow fever and Japanese encephalitis vaccination. The seroprevalence of TBEV antibodies was thus estimated to 0.14% (95% CI: 0.05–0.42).

All persons with specific anti-TBEV antibodies were French citizens and had been working in the forest sector for 20–39 years. Two of them worked in the Franche-Comté region (eastern part of France) and one in Pays-de-la-Loire region (western part). One was 30– 40 years old and two were 50–60 years. These three persons were woodcutters, working more than 20 h per week in a forest environment and felt exposed to tick bites. The one working in Pays-de-la-Loire had also been working in Poitou-Charentes and Centre regions, not known as risk areas for TBEV, and had not travelled to a country endemic for TBEV. All were seronegative for *B. burgdorferi* sl.

## Discussion

For the first time, we estimated the prevalence of *B. burgdorferi* sl antibodies among forestry workers in the northern half of France. In the north-eastern regions, *B. burgdorferi* seroprevalences of forestry workers estimated in 2003 [20] were below those of this study (overall 14.1% vs 21.3%), which suggests an increased exposure to the bacterium. An increase in the LB incidence has also been observed since 2003 [5]. However, there were differences in the application and interpretation of laboratory tests: the immunoblot tests used were different and samples with positive ELISA and borderline immunoblot results were classified as seronegative in 2003. Also, sampling methods differed between the two studies as no randomisation of the study participants and no adjustment for participation were done in 2003. These factors might have contributed to the difference in seroprevalence. Regarding TBEV antibodies, we observed a lower seroprevalence in our study than in the study of 2003 (0.14% vs 2.3%) [20]. This may be attributed to the use of more specific tests as we used two different serological tests with better performance and seroneutralisation, instead of only one ELISA in the study of 2003 [24].

Although we had to discontinue the study in March 2020 due to the COVID-19 pandemic, we were still able to meet the main objectives of comparing seroprevalences of *B. burgdorferi* sl antibodies by region and verify our east–west gradient hypothesis on the seroprevalence. Indeed, a significantly higher seroprevalence was observed in the eastern region (21.3%) while the seroprevalence in the central region was 8.6% and 5.5% in the western. Seroprevalence was also higher in areas with high incidence for LB. Our results on seroprevalence of *B. burgdorferi* sl are consistent with the spatial distribution data on infected ticks [6-10], on the prevalence of tick bites [27] and on the notified incidence of LB in general practice [4,5]. Thus, these results add to the knowledge on the exposure to *B. burgdorferi* sl infection over the last years and on spatial exposure to tick bites in France [27].

However, it was more difficult to correlate seroprevalence estimates for TBEV with other surveillance data on TBE since there was no specific TBE surveillance before 2020. The few seropositive workers in our survey raise the question if the virus is present over a larger area of France than the known endemic regions Alsace and Rhône-Alpes. Our study did not identify any forestry workers seropositive for TBEV in Alsace, known as a hot spot for TBE infection. Two seropositive workers were identified in Franche-Comté region bordering Switzerland where incidence of TBE is higher than in France and recently increased with an unprecedented outbreak in 2019 [11].

Our study also indicates that, as expected, forestry workers are much more frequently exposed to tick bites than the general population. Indeed, according to the French Health Barometer Survey conducted in 2019, 30.2% of the population reported having been bitten by a tick and 5.9% of them in the year preceding the survey [27]. In our study, the prevalence of tick bites was 90.3% and 69.3% in the preceding year. Also, forestry workers felt more exposed to tick bites than the general population. In our study, 87.9% felt exposed to tick bites whereas this proportion was 24.9% among general population in a previous study [27]. Several other studies have also shown an increased risk of LB among professionals working outdoors, particularly in high incidence areas and those working in the forestry sector [16,17]. In our study, forestry workers had a better knowledge about ticks and TBD and were more likely to protect themselves than the general population in a previous study [27].

Seroprevalence studies of *B. burgdorferi* sl from Europe, Central and North America and Asia were summarised in two meta-analyses. In the first meta-analysis, an overall seroprevalence of 25% was estimated among forestry workers [18]. Potential differences in the definition of seropositivity and the serological techniques used complicate the comparison with our data. By selecting only studies in which ELISA and immunoblot techniques were used, seroprevalence for antibodies against *B. burgdorferi* sl in forestry workers ranged from 6.9% in Italy to 34.2% in the south-west of Germany. The second meta-analysis estimated a global seroprevalence of 13.5% among forestry workers in Western Europe, confirmed by western blot [28]. The reported pooled seroprevalence in studies using ELISA confirmed by western blot was lower than in those using ELISA not confirmed by western blot. In our study, we wanted to increase specificity and define seropositivity using ELISA confirmed by western blot as was done in a recent German study [29].

Our results on factors associated with seropositivity were consistent with findings from others [18,29-31]. Seroprevalence was higher in persons having been bitten and increased with the number of tick bites. Higher seroprevalences were observed among forest technicians, rangers and silviculturists which suggests that these workers are particularly exposed to ticks during the forest management and maintenance activities. We also found that time spent in forest is a risk factor as persons spending more time weekly in forest had a higher seroprevalence. However, when we asked about the leisure time habits such as hunting, picking berries and mushrooms and hiking, we did not find any association between these activities and *B. burgdorferi* sl seropositivity (data not shown). Thus, forestry workers seem to be predominantly exposed through their occupational activities. Furthermore, we did not find any association with travel in endemic areas. Seroprevalence studies of the general population, for example of blood donors, could allow estimating the risk increase due to occupational activities and confirm if the forestry professionals are more exposed to tick-borne pathogens than the general population.

Increased age and years in the forestry sector were associated with the presence of anti-*B. burgdorferi* sl antibodies which could be explained by the repeated exposure to tick bites and thus to the bacterium, leading to a repeated stimulation of antibody production. Few studies have focused on the persistence of antibodies. In some studies antibodies could be measured for up to 6 months while others have detected antibodies 1–20 years after the infection. The persistence of detectable antibodies depends on several factors such as clinical presentation, severity and duration of the disease, delay of treatment, genetic factors and diagnostic techniques [32-34]. Therefore, a seropositive result does not allow to date the time point of the infection. Seropositivity reflects both recent and ancient exposures to *B. burgdorferi* sl.

Some studies have investigated associations between seropositivity and type of forest environment and found higher *B. burgdorferi* sl seroprevalences among foresters in deciduous or mixed deciduous/coniferous forest areas than in coniferous forests [18]. These environmental data were not collected in our study.

Only few data about TBEV prevalence in ticks or other wildlife are available in France, mostly from Alsace region [35,36]. Ticks infected with TBEV have been found only in certain risk areas. In other areas in Europe, where TBEV is endemic, studies showed a low prevalence of TBEV in *I. ricinus*, varying from 0.1% to 5% [37]. In our study, three forestry workers were seropositive for anti-TBEV antibodies including one who reported not having worked in a risk area in France during the preceding year. This professional did not mention having worked in other regions more than a year before this study, but we cannot exclude a recall bias, and he might have been exposed in a risk area. Furthermore, this person could also have been exposed to TBEV in a risk area during leisure time. The low prevalence of TBEV found in ticks in France and the higher awareness of forestry workers could explain the low number of professionals seropositive for TBEV. Nevertheless, climate change could, in the future, contribute to an expansion of risk areas into new regions [38]. The recent introduction of mandatory notification of TBE in France will help to follow incidence trends and to identify new geographical risk areas and risk factors. TBEV infection has been a growing public health problem in Europe over the past 20 years, except for countries with intensive vaccination programmes [39]. In France, vaccinations are currently recommended to travellers to rural or forestry endemic areas in Europe from spring to autumn. These recommendations could be re-evaluated in light of future surveillance data or seroepidemiological studies.

The random selection of individuals and the non-response adjustment according to socio-demographic, geographical and professional characteristics allowed limiting selection bias in our study. However, we cannot exclude that the exposure to ticks and the level of information on TBD and its prevention were different between people who agreed to participate and those who did not, leading to a residual selection bias.

## Conclusion

This study confirms that forestry workers constitute a population at risk of tick-borne diseases. We showed the importance of considering exposure to ticks when predicting the risk of infection by *B. burgdorferi* sl and that compliance with preventive measures such as rapid tick removal are protective against the infection. Even if it is not possible to extrapolate these seroprevalence results to the general population, they provide a measure of disease risk and spatial exposure in the population as a supplement to clinical case reporting. The results of this study will be used, with data on the incidence of LB and TBE and a study on knowledge, attitude and practices to target prevention programmes and to map risk areas. This also enables us to justify the allocation of more resources for prevention campaigns for the general population in regions with the highest risks.
